# Characterization of the human T cell response to in vitro CD27 costimulation with varlilumab

**DOI:** 10.1186/s40425-015-0080-2

**Published:** 2015-08-18

**Authors:** Venky Ramakrishna, Karuna Sundarapandiyan, Biwei Zhao, Max Bylesjo, Henry C. Marsh, Tibor Keler

**Affiliations:** Celldex Therapeutics, Inc., Hampton, NJ 08827 USA; FiosGenomics, Edinburgh, EH16 4UX UK; Celldex Therapeutics, Inc., Needham, MA 02494 USA

**Keywords:** Costimulation, CD27, Immunotherapy, T cells, Monoclonal antibody

## Abstract

**Background:**

Clinical targeting of TNFR family of receptors (CD40, CD134 and CD137) with immunostimulatory monoclonal antibodies has been successful in cancer immunotherapy. However, targeting of CD27 with a mAb is a relatively new approach to provide costimulation of immune cells undergoing activation. Thus, activation of human CD27 (TNFRSF7) with a monoclonal antibody (varlilumab) has previously been demonstrated to result in T cell activation and anti-tumor activity in preclinical models, and is currently in early phase clinical trials in patients with advanced malignancies. In this study we used an in vitro system using human peripheral blood T cells to characterize the varlilumab-mediated costimulatory effects in combination with TCR stimulation in terms of phenotypic, transcriptional and functionality changes.

**Methods:**

T cells were isolated from normal volunteer PBMCs using magnetic bead isolation kits and stimulated in vitro with plate bound anti-CD3 Ab (OKT3) and varlilumab or control Ab for 72 h. Activation profiles were monitored by ELISA or Luminex-based testing cytokine/chemokine releases, cell surface phenotyping for costimulatory and coinhibitory markers and CFSE dye dilution by proliferating T cells and Tregs. Changes in gene expression and transcriptome analysis of varlilumab-stimulated T cells was carried on Agilent Human whole genome microarray datasets using a suite of statistical and bioinformatic software tools.

**Results:**

Costimulation of T cells with varlilumab required continuous TCR signaling as pre-activated T cells were unable to produce cytokines with CD27 signaling alone. Analysis of T cell subsets further revealed that memory CD4+ and CD8+ T cells were specifically activated with a bias toward CD8+ T lymphocyte proliferation. Activation was accompanied by upregulated cell surface expression of costimulatory [4-1BB, OX40, GITR and ICOS] and coinhibitory [PD-1] molecules. Importantly, varlilumab costimulation did not activate purified Tregs as measured by cytokine production, proliferation and suppression of dividing non-Treg T cells. Analysis of changes in gene expression during varlilumab stimulation of T cells revealed modulation of pro-inflammatory signatures consistent with cellular activation and proliferation, with the IL-2 pathway showing the highest frequency of gene modulation.

**Conclusions:**

Altogether, the data reveal the requirements and T cell subtype-specific effects of CD27 costimulation, and helps select relevant biomarkers for studying the effects of varlilumab in patients.

**Electronic supplementary material:**

The online version of this article (doi:10.1186/s40425-015-0080-2) contains supplementary material, which is available to authorized users.

## Background

CD27, a member of the TNFR superfamily (TNFRSF7), is constitutively expressed as a costimulatory molecule on naïve, activated and memory T cells, NK and NKT cells, Treg and B lymphocytes. Its expression increases upon T cell activation, and is lost at the fully differentiated effector phase [1]. The ligand (CD70, CD27L) is only transiently expressed in matured DCs and activated T and B cells [2–4]. The CD27-CD70 axis appears to be important in cell survival, maintenance of memory cell function, anti-tumor cellular immunity and autoimmunity [5–9]. Consistent with its role in T cell activation and proliferation, CD27 costimulation improves the function of T cells genetically engineered to express chimeric antigen receptors (CARs) [10]. In humans, the role of CD27 signaling in T cells is complex as it is unclear whether the different lymphocyte subsets that express this molecule are under the control of different transcription factors or in a state of activation or differentiation.

Stimulatory and inhibitory signals are used to control the threshold of T cell activation, survival, cytokine-producing capacity and differentiation in a context dependent manner [11]. A number of the costimulatory molecules are members of the TNFSF/TNFRSF that currently consist of 19 ligands and 29 receptors expressed by non-lymphoid and lymphoid cells [12]. T cells can only translate signals through TNFRSFs in a TCR-dependent manner to support costimulation, coinhibition, proliferation, survival, cytolysis, polarization, expansion of memory effectors and death [13, 14]. Utilizing agonist mAbs to target select TNFR members has proven an effective treatment modality in preclinical cancer models [15–18], which has led to translational clinical studies of agonist mAbs specific for CD40, CD137, and OX40 [19–21].

Although the CD27 pathway has been studied less in this context, a number of studies support that this TNFR member may also be exploited for cancer immunotherapy. Forced expression of CD70 in a transgenic mouse model provides protection against an otherwise lethal challenge of tumor cells by constitutive activation of the CD27 pathway [22]. Furthermore, agonist mAbs specific for mouse CD27 have shown efficacy against syngeneic tumors [23, 24]. The anti-tumor activity of these anti-CD27 mAbs was shown to require IFN-γ and is associated with an increase in effector cells in the tumor [25].

We have previously described the development and characterization of an agonist fully human anti-CD27 mAb, varlilumab (also known by 1F5 or CDX-1127) [26, 27]. However, very little is known about a direct costimulatory function of human T cells with the use of varlilumab. In this study we examine in more detail the conditions of CD27 costimulation of in vitro activated human T cells by varlilumab and show that the two signals of activation must be presented concurrently to support a regulatory role for this agonist anti-CD27 mAb that shapes transcriptional and translational changes manifested as gene expression, pathway activation and biological function. These findings may have direct application to understanding the effects of varlilumab in treated patients, and may clarify its effects as distinct from the larger group of costimulatory mAbs in development for activating immune responses against cancer.

## Methods

### Antibodies

Fully human anti-CD27 mAb (varlilumab; varli) was produced as previously described [25, 26], commercially available monoclonal anti-human CD3ε (OKT3 clone, eBioscience), ultra-LEAF Abs to 4-1BB, CD28, OX40, GITR, superagonist anti-CD28 (CD28SA clone 28.2) and isotype-matched control murine IgG1s were purchased from BioLegend, control hIgG (fully human IgG1, Celldex) and secondary goat anti-human LEAF Ab for crosslinking were purchased from Southern Biotech (Birmingham, AL). Mouse anti-human Abs to CD27 (M-T271), CD3, CD4, CD8, CD45RA, CD45RO (all from BD Biosciences); mouse anti-huCD70 blocking Ab (clone BU.69) was purchased from Abcam (UK).

### Generation of CD70-293 and mock-293 transfectants

A vector containing the cDNA for human CD70 (accession # NM_001252.2) was purchased from Origene (Rockville, MD). The DNA was digested with EcoRI and NotI to release the fragment containing CD70, which was inserted in the Origene vector pCMV6 (catalogue # pS100009). The correct clone was confirmed by restriction digest analysis and sequencing. FS-293 cells (a variant of HEK 293 cell line) were used for transfections with the pCMV-CD70 (293-CD70) or pCMV-mock vector (293-mock) using the Invitrogen (Carlsbad, CA) reagents and following the manufacturer’s protocol. At 48 h post transfection, stable transfectants were selected with G418 (0.5 mg/mL). Cell surface CD70 expression was monitored by flow cytometry and high expressing cell lines were selected for experiments.

### Primary cell preparations and activation protocols

Freshly prepared buffy coats or whole blood were obtained from normal healthy volunteers from a local blood bank (Biological Specialty Corp., Colmar, PA) and shipped within 2 h of collection in heparinized bags or tubes. PBMCs were isolated by standard density gradient centrifugation on Ficoll-Hypaque (Thermo-Fisher). T cell purification was carried out by cell sorting on magnetic beads by negative selection (Human Pan T cell isolation kit; Human Treg isolation kits (CD4/CD25hi/FoxP3 and CD4/CD25hi/CD127dim) and CD3/CD4 or CD3/CD8 isolation kits; Miltenyi-Biotec, Auburn, CA) and used directly for in vitro stimulations. For stimulation studies, multi-well culture plates were dry-coated with various antibodies or antibody (Ab) combinations. Unless otherwise noted, the concentrations of antibodies used for coating were: OKT3 (1.0 μg/ml), varlilumab (10 μg/ml), control hIgG1 (10 μg/ml), CD28SA (5 μg/ml). Purified T cells (1 × 10^5^ T cells/well) were added to wells of a 96 well plate or 1.5 × 10^6^ cells/well of a 24 well plate) and stimulated with Abs for different durations from 24 to 72 h at 37 °C in a humidified CO_2_ incubator. Cells were cultured in RPMI 1640 basal medium supplemented with 10 % heat-inactivated fetal bovine serum, L-Glutamine, and penicillin/streptomycin. Cell-free culture supernatants were obtained by centrifugation and frozen at −20 °C for cytokine and biomarker analysis.

### Analysis of cytokine production

Multi-well culture plates were dry-coated with OKT3 (1.0 μg/ml) and either varlilumab (10 μg/ml) or control hIgG (10 μg/ml) prior to addition of T cells isolated from individual donors. Cell culture supernatants were collected from quadruplicate wells and pool tested for cytokines in duplicate (IFNγ, IL-2, TNFα and IL-13) by ELISA using commercial kits (R&D Systems) or with a multiplexed Luminex bead-based assay (41-plex panel, Eve Technologies, Calgary, Canada). Statistical treatment of all cytokine/chemokine results was performed using Student’s two-tailed paired *t*-test comparing varlilumab costimulation with control stimulation; *p* values ≤ 0.05 were considered significant. The CV of replicate tests was always less than 5 %.

### Cell signaling pathway assay

T cells were stimulated for 72 h with OKT3/varlilumab (Varli), OKT3/hIgG, or OKT3/anti-CD28 in the presence or absence of pathway-specific small molecule inhibitors (Invivogen, San Diego, CA). The inhibitors were present for the duration of the experiment. The signaling pathways were blocked with T cells pretreated with NF-κB (Celastrol; 5 μM), MAPKK/ ERK1/2 (PD98059; 50 μM), PKR (2-Aminopurine; 5 μM), MAPK p38 (SB203580 10 μM), IκBα (BAY11-7082; 5 μM) and JAK2 (AG490; 50 μM). Supernatants were harvested from quadruplicate wells and pooled for analysis of IFNγ production by standard ELISA. All samples were run in duplicates with CV <5 %.

### Gene expression analysis

T cells (1.5 × 10^6^/well in 24 well plate) were stimulated in vitro separately with two different protocols: Set 1 (3 donors) was 72 h of continuous costimulation (varlilumab/OKT3), while Set 2 (4 donors) was 46 h of pre-activation with OKT3 followed by 4 h of costimulation (varlilumab and OKT3 or isotype control and OKT3). After stimulation, cell pellets were snap frozen and processed for RNA extraction (Miltenyi RNA Isolation Kit), QA/QC testing, and hybridization on Agilent Whole Human Genome Oligo Microarrays (8 × 60K, Miltenyi-Biotec, Auburn, CA). Raw data were processed by FiosGenomics (Edinburgh, UK). The data sets were background-corrected and normalized using quantile normalization [28] from the green channel. Statistical analysis was performed between the treatment groups (varlilumab/OKT3 versus human IgG1/OKT3) within each set using hypothesis testing based on empirical Bayes [29] and correcting for false discovery rates using the Benjamini-Hochberg method [30]. A congruence analysis was performed between Set 1 and Set 2 to evaluate any overlap between the two experiments. The evaluation of congruence was performed at both the probe (gene) level as well as the pathway (Gene Ontology/KEGG) level. Differentially expressed genes were called at an adjusted *p*-value <0.05. Heatmaps were generated by calculating the log2 expression mean for each gene and subtracting that from the iso and test data to yield a scale ranging from −2 to +2 or another scale representing actual fold changes unless otherwise noted. Genes were sorted according to log2 fold changes. Heatmaps were created using the MultiExperiment Viewer software (MeV) available in the TM4 Microarray Software Suite [31].

### Accession code

NCBI Gene Expression Omnibus (GEO): microarray data generated in this study are available in the link:

http://www.ncbi.nlm.nih.gov/geo/query/acc.cgi?acc=GSE58267.

### Intracellular cytokine staining (ICS)

ICS was used to define T cell subtypes involved in the cytokine response to varlilumab costimulation. For these studies, CD3+ T cells were preactivated with OKT3 for 46 h followed by 4 h of costimulation (varlilumab/OKT3). The Cytofix/Cytoperm kit (BD Biosciences) was used for ICS. Cells were treated with Brefeldin A (GolgiPlug™) for 4 h, and cytokines were stained with anti-IFNγ mAb after permeabilization. The cell surface markers CD3, CD4, CD8, CD69 and CD45RO were used to define T cell subsets by staining with specific antibodies (BD Biosciences) prior to analysis on a FACSCanto II instrument.

### T cell proliferation assa*y*

T cells (1 × 10^6^/ml) were labeled with 0.5 μM carboxyfluorescein succinimidyl ester (CFSE, Life Technologies, Carlsbad, CA) while rotating for 5 min. T cells (1 × 10^5^ cells/well) were plated onto 96-well plates previously coated with varlilumab or hIgG1 (2 μg/well) plus OKT3 (50 ng/well). Cells were then collected after 3 or 7 days incubation and stained with various surface markers and analyzed by flow cytometry. The fluorochrome-conjugated antibodies (BD Biosciences) were used to stain for cell surface CD8, CD4, 4-1BB, OX40, PD-1, ICOS, CD27 and CTLA-4. For Treg analysis purified CD4 cells were cultured with dry-coated antibodies as described and were fixed and detected using a Treg FoxP3-based phenotyping kit following the manufacturer’s protocol (eBiosciences) and analyzed on a FACSCanto II instrument.

## Results

### Properties of varlilumab costimulation of T cells

**Fig. 1:**
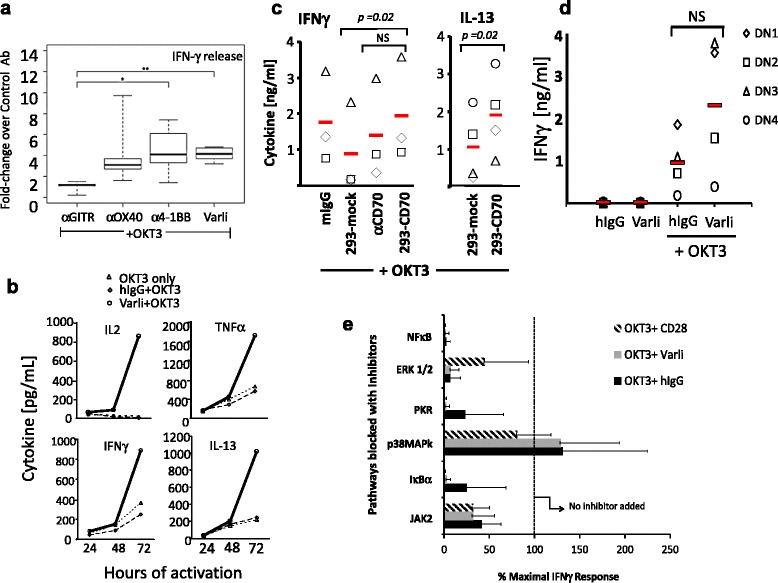
Cytokine induction, kinetics and TCR dependence of anti-CD27 costimulation of T cells by varlilumab or natural ligand (CD70). T cells were stimulated concurrently for 72 h with anti-CD3 (OKT3) and anti-CD27 (varlilumab; varli) antibodies. **a** Boxed whisker plot showing direct comparison of varlilumab stimulation of T cells with that of 4-1BB, OX40 and GITR-specific Abs [*n* = 6]. Statistical comparisons of IFNγ release by the different TNFR stimulations was performed using Welch Two Sample *t*-test; significance is shown as *p ≤ 0.05; **p ≤ 0.01. **b** Cell culture supernatants were tested for Th1/Th2 cytokines at the time points indicated by standard ELISA; representative data of 3 donor samples is shown; **c** T cells were stimulated for 72 h with OKT3 and irradiated 293-CD70 or 293-mock cell lines and assessed for IFNγ and IL-13. Additionally, CD70-dependent IFNγ release was assessed in the presence of a blocking Ab (BU.69) or control Ab (mIgG, left panel; *n* = 3) and IL-13 (right panel; *n* = 4); **d** T cells were first pre-activated for 46 h with anti-CD3 and further stimulated with varlilumab or hIgG or in combination with anti-CD3 for 24 h and tested for IFNγ (*n* = 4); NS, not significant (*p* = 0.09). **e** NFκB signaling by varlilumab-costimulated T cells for 72 h showing differential release of IFNγ in the presence of known pathway-specific inhibitors (top to bottom)- NFκB (Celastrol), ERK1/2 (PD98059), PKR (2-aminopurine), p38 MAPK (SB203580), IκBα (BAY11-7082), and JAK2 (AG490); bars represent mean ± SD (*n* = 4)

We have previously shown that varlilumab can activate human T cells in the context of TCR stimulation, but only when the antibody is plate-bound to provide effective cross-linking [26]. We sought to investigate more thoroughly the properties of varlilumab costimulation through studies that investigated comparison to other costimulatory pathways, kinetics of activation, breadth of the cytokine response and signaling pathways. For these studies we used purified T cells added to plates coated with a suboptimal dose of OKT3 in a 3 day costimulation assay using cytokine production as readout for activation. We initially showed that this method provides effective costimulation through different TNFRSF members- CD27, 4-1BB, OX40 and GITR (Fig. 1a). Analysis of IFNγ responses using T cells from six donors showed comparable costimulation with the CD27, 4–1 BB and OX40 Abs, while a smaller increase was noted with GITR Ab under these assay conditions.

We investigated the kinetics of costimulation for cytokine production under these conditions, and found that 72 h was required to observe a high response to costimulation, and that these kinetics were similar for each cytokine analyzed (Fig. 1b). As shown with a representative donor, varlilumab treatment elicited significant levels of the Th1 cytokines; IFNγ, IL-2, TNFα but also high levels of the Th2 cytokine IL-13 from stimulated cells in a time-dependent and specific manner as shown with the isotype control Ab. To more fully characterize the cytokine response under these conditions, cell culture supernatants were subjected to a cytokine/chemokine analyses on a multiplex platform. The results show a marked increase of Th1-like/inflammatory cytokines and chemokines, some increases in Th2 cytokines (predominantly IL-13 and IL-5) as well as induction of several growth factors such as GM-CSF and CD40L (see Additional file 1: A-C).

We did not initially expect the combined secretion of IL-13 with the Th1 cytokines, and therefore investigated whether a similar pattern would result from CD27 costimulation of TCR-triggered T cells with its cognate ligand, CD70. For these studies, T cells were cultured with irradiated CD70-expressing or mock-transfected cells in wells containing plate-bound OKT3. As shown, similar to stimulation with varlilumab, T cell interaction with CD70-293 cells but not mock-293 cells resulted in release of high levels of IL-13 and IFNγ (Fig. 1c; *p* < 0.05). Specificity of this response was further shown by blocking the response (in 2 out of 3 donors; *p* = 0.2) with an anti-CD70 mIgG (BU.69) but not with an isotype-matched control IgG.

We next addressed the question whether coincident TCR signaling is critical for CD27-mediated costimulation, or would pre-activated T cells be primed to respond to varlilumab even in the absence of TCR signaling. T cells were pre-activated by initial stimulation for 46 h with OKT3 mAb alone and then transferred to wells coated either with varlilumab or hIgG with or without OKT3 mAb. In this format, T cell activation was more rapid with high levels of IFNγ released within 24 h (Fig. 1d). However, T cell activation still required both TCR and CD27 signaling together, since the pre-activated T cells could not be further stimulated with varlilumab alone, emphasizing the requirement of concomitant TCR signaling at the time of CD27 costimulation (*n* = 4; *p* = 0.09). Moreover, replacing TCR signaling (OKT3) with CD28 stimulation or IL-2 did not result in measurable costimulation with varlilumab (data not shown) further demonstrating that complete activation of T cells with varlilumab only ensues with continuous TCR-triggering.

We also explored the effects of various signaling pathway inhibitors on CD27 costimulation of T cells and how they compared to CD28 costimulation using anti-CD28 super agonistic mAb, CD28SA. As shown in Fig. 1e lack of or reduction in IFNγ release by stimulated T cells in the presence of inhibitors revealed that CD27 costimulation is coupled to 4 of 6 pathways tested (NFκB, ERK1/2, PKR and IκBα signaling) thus resembling pathways initiated by CD28 costimulation. In contrast, however, TCR-triggered pathways utilizing p38MAPkinase and JAK2 were less well intercepted with inhibitors suggesting that these nodes are either not playing a critical role or require participation of auxiliary nodes. CD28 costimulation was distinct from that provided by CD27 in that CD28 only showed a partial involvement of ERK1/2. These observations are consistent with the expected pathways involved in co-stimulation with TNFRSF members and show that T cells may utilize canonical or non-canonical NFκB signaling pathways depending on which costimulatory molecules are engaged.

### Molecular footprinting of gene expression changes in CD27 stimulated T cells

**Fig. 2:**
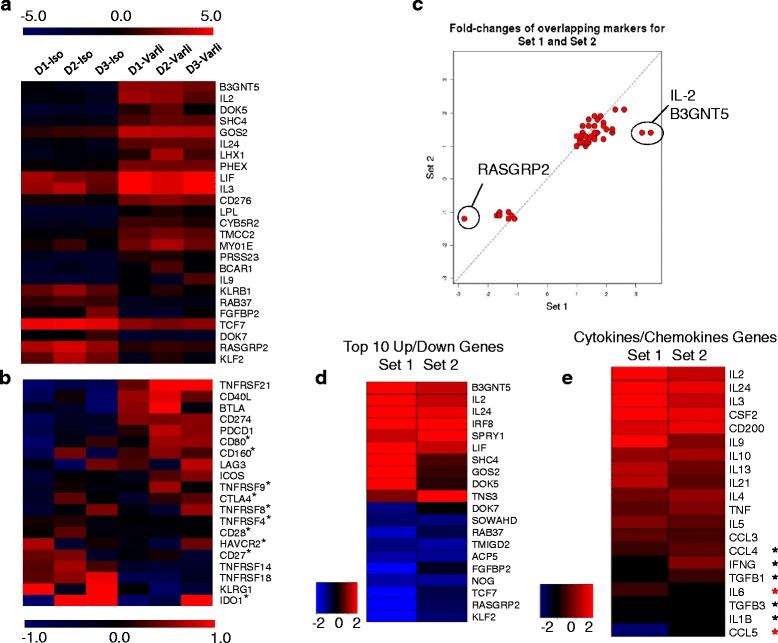
Transcriptome analysis showing changes in gene expression pattern during varlilumab costimulation of T cells. T cells were costimulated continuously for 72 h (Set 1) or first preactivated with anti-CD3 (OKT3) for 46 h and costimulated briefly (4 h) with anti-CD3 + varlilumab or anti-CD3 + control Ab (hIgG; Iso; Set 2). Treated cells were subjected to RNA extraction and hybridization on whole genome arrays as described. **a** Heatmap showing log2 fold-changes as described under Methods shows the top 25 genes in red (upregulated) or blue (downregulated) during varlilumab costimulation compared to isotype IgG stimulated controls in Set 1 protocol (*n* = 3). Log2 expression values were rescaled by calculating a mean log2 expression for each gene and then subtracting that value from each individual gene expression value. This approach effectively centers the gene expression values around zero so that actual fold changes are easily discerned from the heatmap (*p* < 0.05). **b** Select inflammatory panel gene list queried to map transcripts coding for costimulatory (B7 family, TNFRSFs) and coinhibitory (immune checkpoint) molecules post varlilumab stimulation (Set 1). Expression rescaling approach described above was also employed here. Gene symbols with asterisks are not significant (*p* > 0.05). All other genes are significant according to non-adjusted *p* < 0.05. **c** Congruence plot showing varlilumab stimulation resulted in similar gene expression signatures between Set 1 (*n* = 3) and Set 2 (*n* = 4) with 47 overlapped genes. Shown are log2 fold changes with an FDR-adjusted *p* < 0.05. **d** Top 10 genes up/downregulated in response to Set 1 and Set 2 stimulation protocols, Shown are log2 fold changes with an FDR-adjusted *p* < 0.05. **e** Fold-change in transcripts corresponding to cytokines, chemokines and growth factors. Heatmap shows log2 fold change in gene expression comparing Set 1 and Set 2 type varlilumab stimulations. Heatmap representation was generated from an ordered gene list so not all input genes are significant (shown with black asterisks) at pFDR < 0.05. Labels with red asterisks (IL6 and CCL5) were significant in Set 1 with a direction of change being identical in both studies. All genes highlighted in (**b**, **c** and **e**) are significantly different in T cells stimulated with varlilumab compared to isotype control Ab stimulations

We next explored the molecular footprints of interacting genes and pathways following CD27 costimulation. The molecular signature of overall changes in gene expression in activated T cells was undertaken by hybridization of labeled RNA onto commercially available human oligoarrays. T cells from multiple donors were analyzed separately in paired fashion (varlilumab-treated versus control hIgG-treated) using two separate protocols to understand whether there were differences in “early” versus “late” gene expression changes. Thus, T cells were stimulated in a 3 day continuous OKT3/CD27 costimulation (Set 1 or late events) or a pre-activation step with OKT3 alone followed by a OKT3/CD27 costimulation for 4 h (Set 2 or early events). Molecular fingerprints of differentially expressed genes are visualized with heatmaps showing log2 fold-changes in gene expression patterns among top 25 genes up- or down-regulated between varlilumab-stimulated (D1-D3 Varli) versus control-stimulated (D1-D3 Iso) at 72 h (Set 1 Fig. 2a). Next we queried a list of highly enriched statistically significant genes associated with inflammatory signaling pathways. These are gene transcripts coding for TNFRSFs, costimulatory (CD28/CTLA-Ig family) and coinhibitory (immune checkpoint) molecules (Set 1, Fig. 2b). A heatmap representation of this dataset shows some gene transcripts were clearly upregulated (DR6, CD160, BTLA, CD40LG, PD-1, PD-L1, B7.1, CTLA-4, LAG-3, 4-1BB etc.) while other gene transcripts were downregulated (CD27, CD28, KLRG1, OX40, GITR, LIGHT etc.) following 72 h of varlilumab stimulation. Furthermore, genes represented in this heat map were derived from an ordered genes-of-interest (GOI) list and therefore contain significant as well as non-significant gene signatures.

**Fig. 3:**
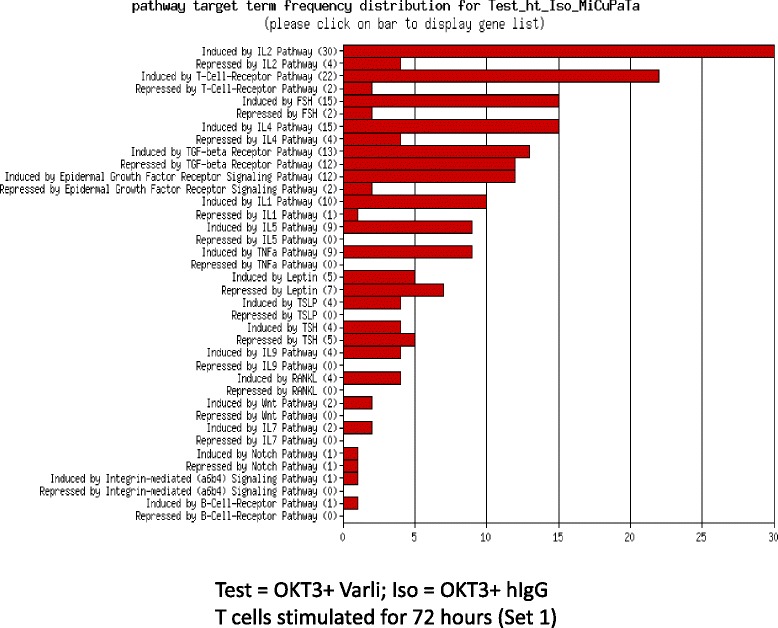
Pathways differentially affected by varlilumab costimulation. A bar chart showing the frequency of representative categories within the gene list (Varlilumab/Test versus Control/Iso/ hIgG_Stringent DGA) with functional grouping. Size of bars denotes the number of genes/targets populating the pathway and independent of the importance of any pathway relative to other pathways. Data were curated on Miltenyi-Biotec’s database of annotated and enriched pathway targets. Numbers in parenthesis refer to number of genes/targets either induced (upregulated) or repressed (downregulated) in the pathway(s) relative to control. DGA, Differential Gene Expression Analysis

We next compared the two varlilumab stimulation protocols (Set 1 and Set 2) to see if these methods were influencing specific or common gene expression profiles. Congruence analysis was performed by evaluating the overlap between Set 1 (469 genes) and Set 2 (82 genes). Overlaps were only considered if the fold changes were also in the same direction. In total, 47 genes overlapped between the two sets. This is significantly higher than expected by chance (*p* = 1.358e-70) from a hypergeometric test. With respect to fold changes these are generally quite consistent between the two sets as they cluster around the diagonal. The main exceptions are IL2 and B3GNT5 which are considerably higher in Set 1 as well as RASGRP2 being considerably lower in Set 1 compared to Set 2 when compared to control-stimulation (Fig. 2c, Additional file 2). Enriched KEGG pathways were also evaluated for overlaps across the two sets, with 33 pathways in Set 1 and 19 pathways in Set 2. A total of 8 KEGG pathways overlapped (Tables 1, 2 and 3; Additional file 2). This is significantly higher than expected by chance (*p* = 0.001943). In contrast, while B3GNT5 a Golgi-resident aminotransferases involved in mucin-type glycan biosynthesis was upregulated, the calcium regulated T cell receptor pathway associated RAS guanyl releasing protein 2 (RASGRP2) was suppressed. Additional analysis included summarized fold-change gene expression patterns for seven donors subjected to Set 1 and Set 2 treatments over Iso controls. Thus, log2 expression data significant at *p* < 0.05 were generated and mapped to list the top 10 genes regulated up/down (Fig. 2d). In addition, log2 fold changes and scaled expression of cytokine-chemokine gene signatures were mapped from a GOI list and contain both significant and non-significant gene signatures (Fig. 2e). Analysis of differentially expressed enriched gene categories and curated pathways showed IL-2 pathway to be highly populated with gene targets compared to other signaling pathways (Fig. 3) of which substantially greater number of genes to be induced rather than repressed (Table 4).

**Table 1:** KEGG pathways unique to set 1

Pathway ID	Pathway name	Direction
5146	Amoebiasis	Up
5100	Bacterial invasion of epithelial cells	Up
3320	PPAR signaling pathway	Up
512	Mucin type O-Glycan biosynthesis	Up
5416	Viral myocarditis	Down
5332	Graft-versus-host disease	Down
5322	Systemic lupus erythematosus	Down
5150	Staphylococcus aureus infection	Down
5145	Toxoplasmosis	Down
5144	Malaria	Down
5140	Leishmaniasis	Down
4940	Type I diabetes mellitus	Down
4916	Melanogenesis	Down
4710	Circadian rhythm - mammal	Down
4640	Hematopoietic cell lineage	Down
4612	Antigen processing and presentation	Down
4514	Cell adhesion molecules (CAMs)	Down
4145	Phagosome	Down
4144	Endocytosis	Down
4062	Chemokine signaling pathway	Down

**Table 2:** KEGG pathways unique to set 2

Pathway ID	Pathway name	Direction
5332	Graft-versus-host disease	Up
5145	Toxoplasmosis	Up
5144	Malaria	Up
5143	African trypanosomiasis	Up
5142	Chagas disease (American trypanosomiasis)	Up
4940	Type I diabetes mellitus	Up
4664	Fc epsilon RI signaling pathway	Up
4640	Hematopoietic cell lineage	Up
524	Butirosin and neomycin biosynthesis	Up
4350	TGF-beta signaling pathway	Down
740	Riboflavin metabolism	Down

**Table 3:** KEGG pathways overlapping set 1 and set 2

Pathway ID	Pathway name	Direction
5330	Allograft rejection	Up
5320	Autoimmune thyroid disease	Up
5310	Asthma	Up
4672	Intestinal immune network for IgA production	Up
4660	T cell receptor signaling pathway	Up
4630	Jak-STAT signaling pathway	Up
4060	Cytokine-cytokine receptor interaction	Up
5323	Rheumatoid arthritis	Down

**Table 4:** IL-2 pathway genes modulated by Varlilumab stimulation of T cells

Gene Symbol	Description
Induced pathway genes	
CD40LG	CD40 Ligand
IL1RAP	Interleukin 1 receptor accessory protein
IL13	Interleukin 13
ADD2	Adducin 2 (beta)
CTNNA1	Catenin (cadherin-associated protein) alpha 1
TERT	Telomerase reverse transcriptase
LAIR2	Leukocyte-associated Ig-like receptor 2
MYC	v-myc Myelocytomatosis viral oncogene homolog (avian)
LIF	Leukemia inhibitory factor
DUSP6	Dual specificity phosphatase 6
OSM	Oncostatin M
CSF2	Colony stimulating factor 2 (granulocyte-macrophage)
IER3	Immediate early response 3
CSF1	Colony stimulating factor 1 (macrophage)
NTRK1	Neurotrophic tyrosine kinase receptor type 1
NFIL3	Nuclear factor, interleukin 3 regulated
IL5	Interleukin 5
GZMB	Granzyme B (granzyme 2, CTL-associated serine esterase 1
XCL1	Chemokine (C motif) ligand 1
IL6	Interleukin 6 (interferon beta 2)
IL10	Interleukin 10
CD300A	CD300a molecule
IL9	Interleukin 9
PRSS23	Protease serine 23
PHLDB2	Pleckstrin homology-like domain, family B, member 2
Repressed pathway genes	
CXCR5	Chemokine (C-X-C motif) receptor 5
MYC	v-myc Myelocytomatosis viral oncogene homolog (avian)
APP	Amyloid beta (A4) precursor protein
IL24	Interleukin 24

### CD27 costimulation supports proliferation and cytokine production from specific T cell subsets

**Fig. 4:**
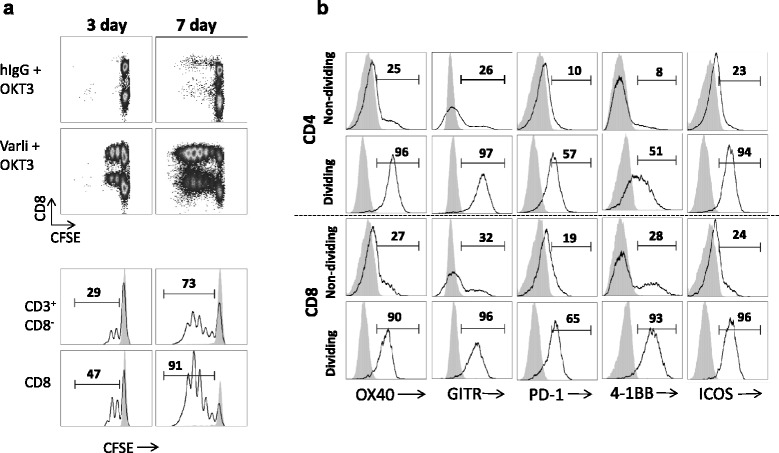
Induced proliferations of T cell subsets and coexpression of costimulatory and coinhibitory receptors (immune checkpoint molecules). Differences in short term (3 day) or long term (7 day) T cell cultures undergoing stimulations with varlilumab or control antibody (hIgG) show more CD8+ cells (**a**) entering the division cycle in the varlilumab-treated group. Numbers in the histograms refer to the percent of indicated cells (CD8+ or CD3+CD8-) that are dividing as reflected by CFSE staining (**a** bottom panel). **b** CD27+ proliferating CD4+ and CD8+ T cells were stained with antibodies to additional cell surface markers representing costimulatory (GITR, OX40, ICOS and 4-1BB) and coinhibitory (PD-1) molecules. Dividing CD8+ and CD4+ T cells show marked upregulation of costimulatory and coinhibitory molecules relative to non-dividing cells. Shaded histograms represent T cells stimulated with control antibody and anti-CD3 (OKT3). Numbers in the histograms refer to percent of T cells (dividing or non-dividing CD8 or CD4 cells) that are positive for the indicated costimulatory or coinhibitory marker (**b**)

**Fig. 5:**
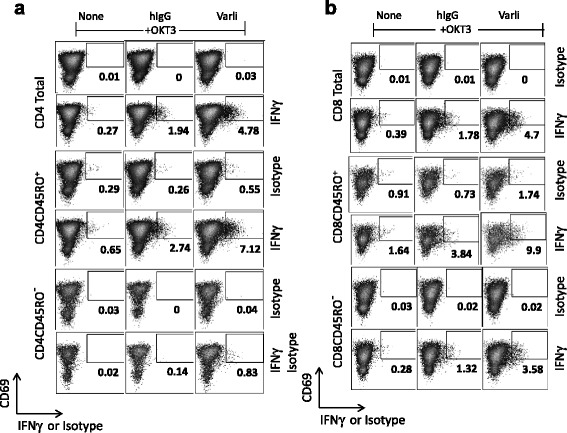
Early detection of cytokines by varlilumab costimulated T cell subsets. T cells were preactivated with OKT3 for 46 h and post activated for 4 h with OKT3+ varlilumab or OKT3+ hIgG. Intracellular staining for IFNγ response in different T cell subpopulations (**a**) Naïve CD4+ (CD45RO-) and memory (CD45RO+) and (**b**) naïve CD8+ (CD45RO-) and memory (CD45RO+) T cells. Cells were co-stained for the activation marker CD69. Numbers in individual plots refer to percent IFNγ+ cells

Functional analysis of T cells co-activated by TCR and CD27 was extended to study proliferative responses and intracellular cytokine staining. CFSE dye-labeled T cells showed a remarkable level of proliferation specifically in response to varlilumab costimulation with the great majority of cells undergoing division by day 7 of culture (Fig. 4a). Due to the production of IL-2 and other cytokines, it is likely that some of the proliferation observed at day 7 is due to indirect effects rather than through direct CD27 costimulation. Interestingly, varlilumab appeared to preferentially stimulate CD8 T cells relative to CD4 T cells (47 % CD8 dividing cells versus 29 % CD4 cells at day 3 and 91 % dividing CD8 T cells versus 73 % CD4 T cells at day 7). The T cells costimulated with varlilumab for 3 days were further characterized for expression of costimulatory and inhibitory molecules that are characteristic of activated T cells (Fig. 4b). Thus, significant upregulation of OX-40, GITR, PD-1, 4-1BB and ICOS was observed on both CD4 and CD8 populations of T cells and preferentially on the dividing cells, consistent with an activated T cell phenotype. We further investigated the T cell subpopulations most responsive to CD27 costimulation with varlilumab. For these studies we pre-activated T cells with OKT3 in order to observe the early response to CD27 costimulation (4 h) by ICS (Fig. 5a and b). Among CD4 T cells, we detected a significant increase in the number of CD69+ IFNγ+ primarily in CD45RO+ (memory) cells. Among CD8 T cells, both CD45RO+ and CD45RO^–^ cells showed an increase in IFNγ production.

**Fig. 6:**
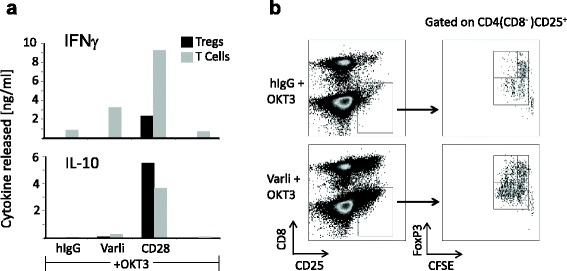
Conventional T cells, but not CD4+CD25hiFoxP3+ (Tregs), can be stimulated by varlilumab to produce cytokines and proliferate. Magnetically isolated Tregs (CD4+/CD25hi/FoxP3+) were compared with CD3+ T cells from the same donor **a** for their response to stimulation with OKT3/varlilumab, OKT3/CD28 or OKT3/control IgG; and **b** CFSE-labeled OKT3-activated and varlilumab stimulated pan T cells were first gated on CD4+CD25+ subset, this subset was then plotted by CFSE and FoxP3. Rectangular gates were drawn to indicate FoxP3- (lower rectangular), FoxP3+ non-dividing (upper-right rectangular), and FoxP3+ dividing (upper-left rectangular), subsets

Tregs are known to express CD27, but the role of CD27 costimulation on these cells and their function is unknown. To this end, we explored the effect of CD27 costimulation on Tregs relative to other T cells. As shown in Fig. 6a purified Tregs exposed to 3 days of costimulation with varlilumab and OKT3 did not result in detectable level of IFNγ or IL-10 secretion. However, costimulation using OKT3 and anti-CD28 showed the capacity of the Treg to produce large amounts of IL-10 and to a lesser extent IFNγ. Importantly, the conventional T cells (i.e. non-Tregs) from the same donors showed the expected high level of IFNγ production and low IL-10 secretion upon CD27 costimulation. We also examined proliferation of the Treg subset by flow cytometry, and did observe some proliferation among Tregs during CD27 costimulation (Fig. 6b upper left gate). However, this was relatively low compared to the FoxP3^−^ T cell populations (lower gate), and may be the result of IL-2 production by activated non-Tregs.

## Discussion

The TNFR superfamily of proteins has gained considerable attention in the last decade with a surge in development of immunomodulatory biologics to treat cancer, infectious diseases and autoimmunity. Targeting signaling molecules to enhance anti-cancer immunity has focused on two approaches: (a) activating costimulatory molecules with agonistic Abs directed to T cell surface molecules- CD27, CD40, OX40 and CD137; or (b) blockade of inhibitory pathways with monoclonal Abs directed to cell surface CTLA-4, PD-1/PD-L1, TIM-3, BTLA and LAG-3 [32]. From a clinical perspective, more progress has been achieved with blockade of inhibitory pathways than with the agonist mAbs. This is at least partially due to the fundamental safety challenges of triggering potent costimulatory pathways, which was exemplified by the experience using an anti-CD28 mAb [33], and dose-limiting toxicities for the development of mAbs to CD40 and CD137 have also been reported [19, 20]. To implement effective costimulatory strategies in the clinic, further characterization and understanding of these agents and their effects is required.

In the case of CD27, the development of mAbs with costimulatory activity is further complicated by data that support the potential anticancer effects of antagonizing this pathway. For example, overexpression of CD70 by tumor cells has been shown to promote T cell dysfunction or apoptosis through CD27 signaling in vitro [34, 35]. However, this effect was overridden by the immune activating properties of CD27 signaling when tested in tumor models [36]. In addition, CD27 signaling in Treg (discussed further below) has been implicated as a means of tumor escape [37], further emphasizing the need to fully characterize the functional activity of CD27 agonist mAbs. In this study we sought to better characterize the costimulatory properties of varlilumab, the first anti-CD27 mAb to enter clinical trials for treatment of lymphomas and solid tumors. Our analysis was mainly conducted on T cells isolated from healthy individuals, cultured under controlled conditions, and demonstrated a robust activation when varlilumab is combined with TCR stimulation. This study did not analyze other CD27-expressing immune cells such as NK or B cell subsets. The majority of circulating NK cells does not express significant levels of CD27, and our preliminary results suggest that varlilumab does not have a significant effect on circulating B cells (data not shown).

Initially, we compared varlilumab to commercially available Abs to 4-1-BB, GITR and OX40 in the ability to elicit IFNγ production under one standardized condition. Thus, T cell CD27 costimulation with varlilumab was comparable to anti-4-1BB, anti-OX40 costimulations but to a lesser extent with anti-GITR Abs. This study was not meant as a comprehensive comparison of these related costimulatory molecules, but rather as a demonstration that the conditions used in our study are relevant to other related TNFRSF members. Important to the mechanism and safety of varlilumab, we show that TCR triggering must be provided simultaneously with CD27 activation, even in pre-activated T cells in order to result in effective stimulation as measured by cytokine secretion.

The cytokine response to varlilumab costimulated T cells was characterized to include high levels of Th1 cytokines (IFNγ, TNFα, and IL-2), and also significant induction of some Th2 cytokines, dominated by IL-13. This pattern was not unique to costimulation of CD27 with varlilumab, as we observed similar IFNγ and IL-13 secretion from T cells activated with the CD27 ligand, CD70. A similar pattern of Th1 cytokines and IL-13 secretion has been reported for a CD137 agonist mAb, and thus may represent a general feature of TNFR costimulation. In this study IL-13 secretion has been thought to provide a feedback mechanism to dampen excessive inflammation [38].

To understand whether CD27 downstream events of cytokine production were dependent on canonical or alternate NFkB signaling, T cells were stimulated in the presence of pathway–specific inhibitors. These experiments revealed that IFNγ production was compromised in the presence of JAK2, ERK1/2 and PKR signaling inhibitors which somewhat overlapped with CD28 signaling and consistent with previous reports on CD27 signaling pathways [39, 40]. We also explored the changes in gene expression and signaling pathways following CD27 costimulation. Significant regulation of other TNFRSF members, cytokines and other immune related markers was observed, and genes in the IL-2 signaling pathway were most significantly affected consistent with studies previously reported by Peperzak et al. [41]. While the microarray technology allows for a rapid high throughput and high content analysis of gene expression in in vitro stimulated cells it only provides a snapshot of subtle/marked changes in transcriptional levels of genes and targets implicated in functional cell signaling pathways. The information obtained must therefore be interpreted with caution as it is not uncommon to find a lack of correspondence between transcriptional events and protein expression.

To gain insight into specific populations of T cells responding to CD27 costimulation, we used flow cytometry to measure proliferation and cytokine production coupled with T cell markers. Varlilumab costimulation induced potent proliferation of both CD8+ and CD8- T cells, but its effect was greatest on the CD8+ T cells. This appears to be due to the responsiveness of both CD45RO+ and CD45RO- subsets of CD8 T cells, while only those CD4 T cells with the memory phenotype were stimulated under these conditions. The observed differences were not likely due to differential CD27 surface expression among T subsets since we observed lowest basal CD27 expression on the CD45RO+ cells (see Additional file 3). However, other factors such as the intrinsic capacity of these cell populations to become activated in vitro and the variable expression of inhibitory receptors may also account for the observed differential proliferative capacity of CD8s versus CD4s. The fate of cells activated by anti-CD3 and a costimulatory molecule often is accompanied by upregulation of accessory molecules that either amplify or dampen the existing response. Interestingly, we have observed extended coexpression of 4-1BB, GITR, OX40, ICOS, CTLA-4 and PD-1 believed to be associated with CD27 triggering in presence of TCR triggering. The discordance of some of these changes with the gene expression data is unclear at present but suggest a more complex network of genes being co-regulated that could affect kinetics of biomarker availability on the cell surface.

CD27 is expressed constitutively on human Tregs [42] and CD27 co-stimulation in the thymus rescues developing Treg cells from apoptosis and thereby promotes Treg generation [43]. CD27 signaling has also been implicated in Treg expansion in mice] where increased numbers of Tregs in tumor infiltrating lymphocytes, spleens and nodes were reported in CD27(+/+) wild type mice relative to CD27(−/−) mice suggesting that CD27-CD70 signaling increases the number of Tregs [37]. Because a comparison of mice completely lacking CD27 compared to those simply expressing CD27 is not necessarily representative of the effects of a particular agonist anti-CD27 mAb, we therefore explored the effect of varlilumab costimulation on proliferation and cytokine production on purified Treg cells. We showed that cytokine induction and stimulation of proliferation by varlilumab were much lower for Tregs than for conventional T cells, and much lower than that induced by anti-CD28 as well. This suggests that CD27 and CD28 may follow divergent pathways in Treg stimulation and is consistent with other reports on Treg costimulation requirements [44, 45]. Our in vitro results using human T cells are consistent with mouse tumor model studies in which an agonist anti-mouse CD27 mAb (AT124-1) reduced the numbers of FoxP3-expressing cells within tumors but had no effect on the suppressor activity on a per cell basis [25]. Our in vitro results are also consistent with our early clinical results [46] in which treatment with varlilumab leads to a significant and roughly 50 % reduction in Tregs in the peripheral circulation. While this does not preclude the potential benefit of antagonizing the CD27 pathway as has been proposed by others [47], the cumulative data with our agonist mAb, varlilumab, does not support the occurrence of unwanted Treg activation.

## Conclusions

Altogether, this study documents the requirements, kinetics and breadth of the in vitro T cell activation signature with varlilumab costimulation through CD27. Consistent with earlier studies, the data demonstrate a tightly regulated response that includes both immune potentiating and immune regulating changes. These molecular and cellular patterns should predict biomarkers that may be impacted in patients treated with varlilumab. Correlations of these markers with the safety and clinical benefit from varlilumab treatment is ongoing and will be important to better understand which features of CD27 costimulation are most relevant to outcomes seen in patients.

## Additional files

Additional file 1:
**Multiplexed biomarker analysis showing modulation of Th1/Th2 cytokines, chemokines and growth factors post varlilumab stimulation.**
Additional file 2:
**Genes and pathways of varlilumab stimulation analysis using Venn charts that show relationship of genes and cell signaling pathways in T cells.**
Additional file 3:
**Baseline CD27 expression levels in T cell subsets- naïve, memory and circulating CD4/CD25hi/CD127dim Treg population in normal volunteer peripheral blood. **

## Abbreviations

Ab: Monoclonal antibody
OKT3: Anti-CD3 antibody clone
Varli: Anti-CD27 Ab (varlilumab)
hIgG: Human IgG or Isotype Control Ab
G418: Geneticin sulphate
FDR: False discovery rate
KEGG: Kyoto encyclopedia of genes and genomes
