# Supplementary material for: Characterization of the human T cell response to in vitro CD27 costimulation with varlilumab
Source: J Immunother Cancer. 2015 Aug 18;3:37. doi: 10.1186/s40425-015-0080-2 (PMC4619281; doi:10.1186/s40425-015-0080-2)
Supplement: Additional file 1: — Multiplexed biomarker analysis showing modulation of Th1/Th2 cytokines, chemokines and growth factors post varlilumab stimulation. [file 40425_2015_80_MOESM1_ESM.pptx]

## Slide 1
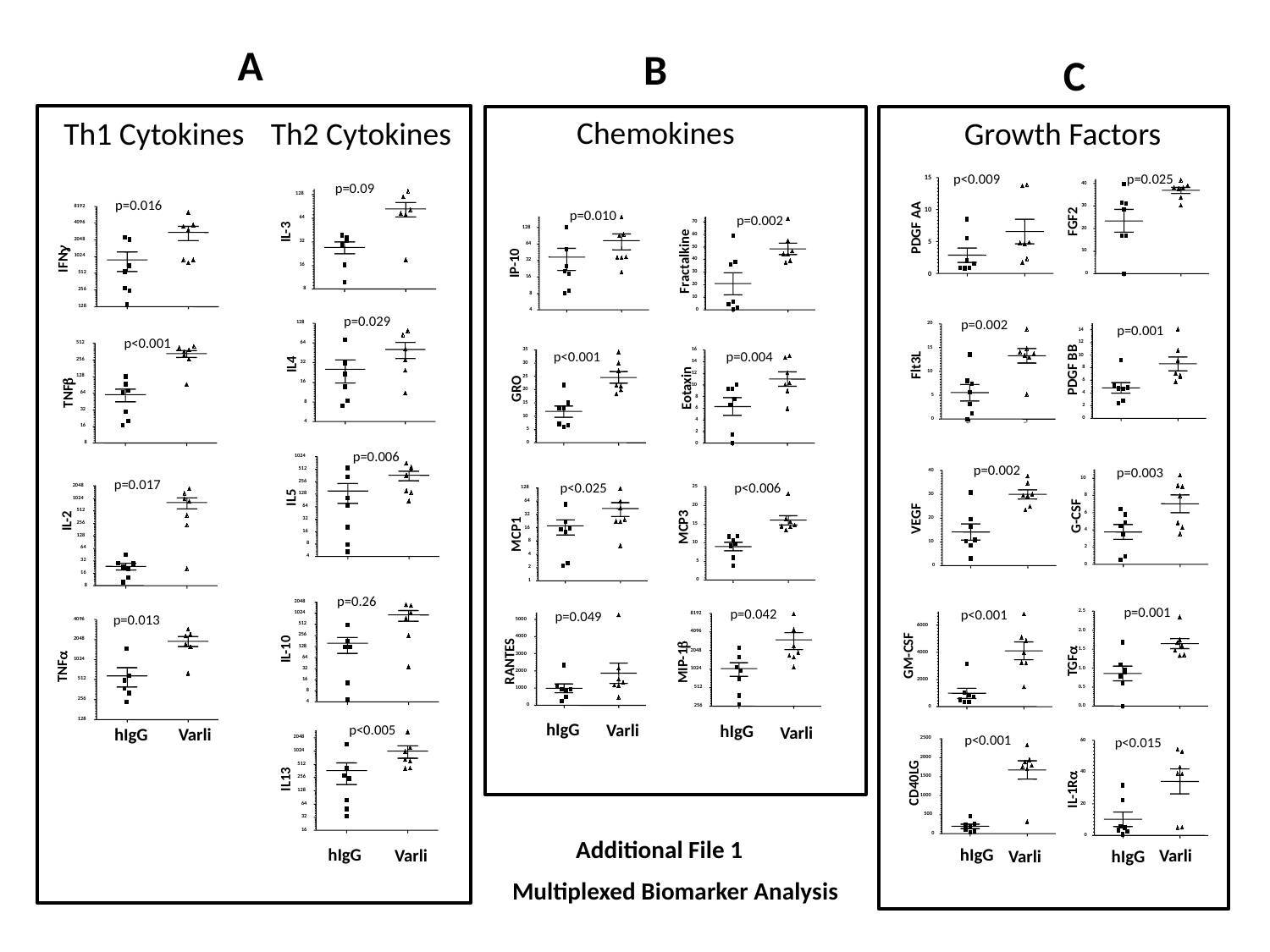

A
B
C
Th1 Cytokines
p=0.016
8192
4096
2048
IFNg
1024
512
256
128
p<0.001
512
256
128
TNFb
64
32
16
8
p=0.017
2048
1024
512
IL-2
256
128
64
32
16
8
p=0.013
4096
2048
1024
TNFa
512
256
128
hIgG
Varli
Th2 Cytokines
p=0.09
128
64
IL-3
32
16
8
p=0.029
128
64
IL4
32
16
8
4
p=0.006
1024
512
256
IL5
128
64
32
16
8
4
p=0.26
2048
1024
512
256
IL-10
128
64
32
16
8
4
p<0.005
2048
1024
512
IL13
256
128
64
32
16
hIgG
Varli
Chemokines
p=0.010
128
64
IP-10
32
16
8
4
p=0.002
70
60
50
Fractalkine
40
30
20
10
0
16
p=0.004
14
12
10
8
6
4
2
0
Eotaxin
35
p<0.001
30
25
GRO
20
15
10
5
0
p<0.025
128
64
32
16
MCP1
8
4
2
1
p<0.006
25
20
MCP3
15
10
5
0
p=0.042
8192
4096
2048
MIP-1b
1024
512
256
p=0.049
5000
4000
3000
RANTES
2000
1000
0
hIgG
Varli
hIgG
Varli
Growth Factors
p=0.025
40
30
FGF2
20
10
0
p<0.009
15
10
PDGF AA
5
0
p=0.002
20
15
Flt3L
10
5
0
hIgG
1F5
p=0.001
14
12
10
PDGF BB
8
6
4
2
0
p=0.002
40
30
VEGF
20
10
0
p=0.003
10
8
G-CSF
6
4
2
0
p=0.001
2.5
2.0
1.5
TGFa
1.0
0.5
0.0
p<0.001
6000
GM-CSF
4000
2000
0
p<0.001
2500
2000
1500
CD40LG
1000
500
0
p<0.015
60
40
IL-1Ra
20
0
hIgG
Varli
Varli
hIgG
Additional File 1
Multiplexed Biomarker Analysis

## Slide 2
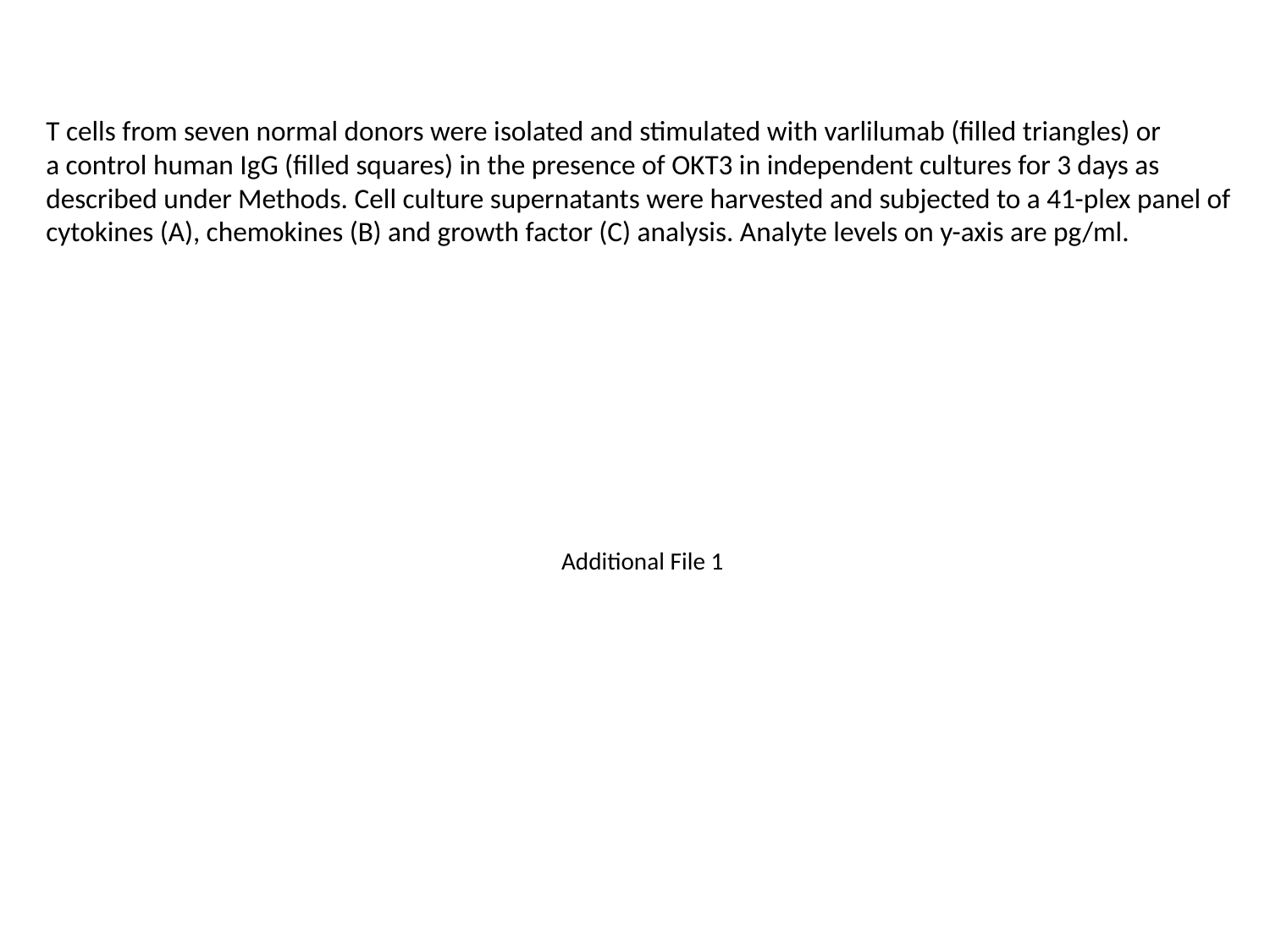

T cells from seven normal donors were isolated and stimulated with varlilumab (filled triangles) or
a control human IgG (filled squares) in the presence of OKT3 in independent cultures for 3 days as
described under Methods. Cell culture supernatants were harvested and subjected to a 41-plex panel of
cytokines (A), chemokines (B) and growth factor (C) analysis. Analyte levels on y-axis are pg/ml.
Additional File 1
