# Supplementary material for: Characterization of the human T cell response to in vitro CD27 costimulation with varlilumab
Source: J Immunother Cancer. 2015 Aug 18;3:37. doi: 10.1186/s40425-015-0080-2 (PMC4619281; doi:10.1186/s40425-015-0080-2)
Supplement: Additional file 2: — Genes and pathways of varlilumab stimulation analysis using Venn charts that show relationship of genes and cell signaling pathways in T cells. [file 40425_2015_80_MOESM2_ESM.pptx]

## Slide 1
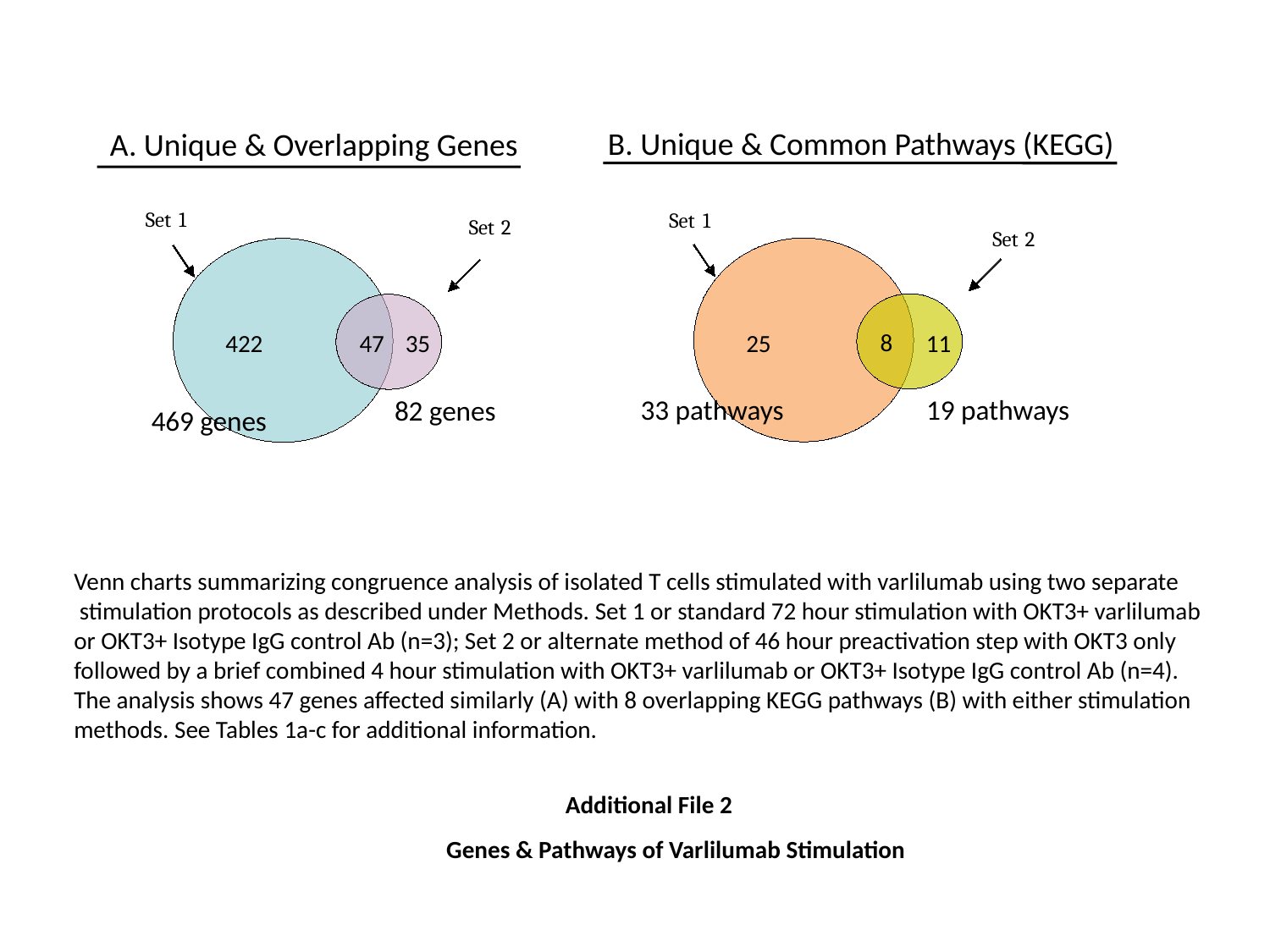

B. Unique & Common Pathways (KEGG)
Set
1
Set
2
8
25
11
33 pathways
19 pathways
A. Unique & Overlapping Genes
Set
1
Set
2
47
422
35
82 genes
469 genes
Venn charts summarizing congruence analysis of isolated T cells stimulated with varlilumab using two separate
 stimulation protocols as described under Methods. Set 1 or standard 72 hour stimulation with OKT3+ varlilumab
or OKT3+ Isotype IgG control Ab (n=3); Set 2 or alternate method of 46 hour preactivation step with OKT3 only
followed by a brief combined 4 hour stimulation with OKT3+ varlilumab or OKT3+ Isotype IgG control Ab (n=4).
The analysis shows 47 genes affected similarly (A) with 8 overlapping KEGG pathways (B) with either stimulation
methods. See Tables 1a-c for additional information.
Additional File 2
Genes & Pathways of Varlilumab Stimulation
