# Supplementary material for: Characterization of the human T cell response to in vitro CD27 costimulation with varlilumab
Source: J Immunother Cancer. 2015 Aug 18;3:37. doi: 10.1186/s40425-015-0080-2 (PMC4619281; doi:10.1186/s40425-015-0080-2)
Supplement: Additional file 3: — Baseline CD27 expression levels in T cell subsets- naïve, memory and circulating CD4/CD25hi/CD127dim Treg population in normal volunteer peripheral blood. [file 40425_2015_80_MOESM3_ESM.pptx]

## Slide 1
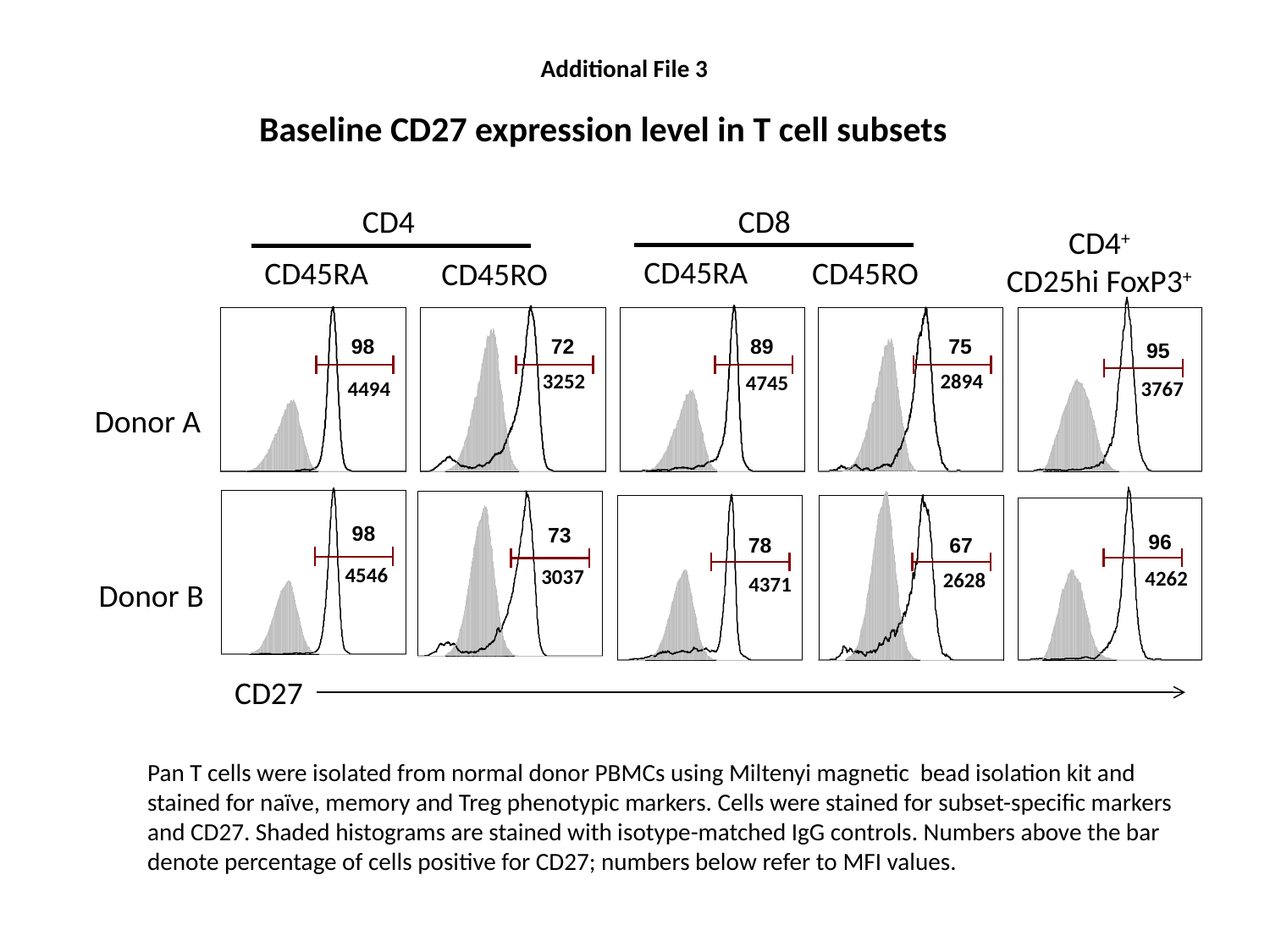

Additional File 3
Baseline CD27 expression level in T cell subsets
CD8
CD4
CD45RA
CD45RA
 CD45RO
CD45RO
CD4+
CD25hi FoxP3+
Donor A
Donor B
CD27
Pan T cells were isolated from normal donor PBMCs using Miltenyi magnetic bead isolation kit and stained for naïve, memory and Treg phenotypic markers. Cells were stained for subset-specific markers and CD27. Shaded histograms are stained with isotype-matched IgG controls. Numbers above the bar denote percentage of cells positive for CD27; numbers below refer to MFI values.
